# Dataset of carabid beetles in Southern Dolomites from 1983 to 1996

**DOI:** 10.3897/BDJ.13.e148207

**Published:** 2025-04-15

**Authors:** Roberto Pizzolotto, Fabiola Durante

**Affiliations:** 1 Università della Calabria, Rende (CS), Italy Università della Calabria Rende (CS) Italy

**Keywords:** Alps, Darwin Core, GBIF, Italy, soil fauna

## Abstract

**Background:**

The Carabid beetles are a key group for assessing ecological characteristics of natural environments because they play a role as bioindicator organisms suitable for both theoretical and applied studies. Moreover, they are one of the few Coleoptera orders with numerous quantitative ecological studies conducted using consistent methodologies in the Dolomite environments (Eastern Italian Alps). In this paper, natural and semi-natural habitats located at mid- and high-altitude located in the Southern Dolomites were studied, specifically the populations of carabids living in alpine grasslands and active scree-slopes from 1300 m a.s.l. to 2160 m a.s.l.

**New information:**

This dataset advances the initiative of adopting a formal structure for carabid beetle datasets and promotes open access to such data. This publication specifically targets historical data published in grey literature, written in Italian or published as technical reports not available online. The Darwin Core standard was used to create the datasets and tables have been made available through the GBIF portal (the Global Biodiversity Information Facility). This ensures the data adhere to the FAIR principles (Findable, Accessible, Interoperable, Reusable), aligning with current best practices in scientific research.

## Introduction

The aim of this dataset is to increase our understanding of carabid beetle communities inhabiting mountain environments in the Eastern Alps. The first contribution of this study is to make datasets available to the scientific community via the GBIF portal, making them freely accessible and compliant with FAIR principles. Additionally, we gave FAIR quality to data previously limited to grey literature or Italian-language publications (or both), therefore inaccessible to the international research community (Brandmayr and Pizzolotto 1989, Pizzolotto and Lasen 1997). The publication of open data promotes an ethical approach to ecological research from a social and scientific point of view by avoiding waste of public funds on repeating the same research and by avoiding the need for duplicate sampling studies in identical (or similar) environments by other researchers (Pizzolotto 2024).

### Object of study

The main object of this study is historical data on carabid beetles, sourced from grey literature written in Italian and from technical reports not accessible online. Most of the material is stored in the collection of the Dept. B.E.S.T. of the Calabria University (Italy). The focus on these organisms is due to their well-documented biology and ecological significance, both globally and specifically in the Italian Alps (Turin et al. 2003,Vanbergen et al. 2010, Gobbi et al. 2010, Chamberlain et al. 2020, Gobbi 2020, Gobbi et al. 2021, Steinwandter et al. 2022, Turin et al. 2022, Steinwandter and Seeber 2023, Dallas et al. 2023). The Carabidae is a family of insects, whose species, in mountain environments, live at ground level, mostly as predators and mostly with an annual life cycle. Relationships between species grouping and the main biotic and abiotic factors characterising the environments in which they live has been documented (Harvey et al. 2008, Duflot et al. 2014, Turin et al. 2022, Lange et al. 2023, Liu et al. 2024, Weiss et al. 2024, Zitzmann et al. 2024). Carabids are an ideal study family for environmental assessment and planning, as they play an important role in many ecosystems: they feed on small invertebrates and are part of the diet of amphibians, reptiles, birds and small mammals; considering their size and biomass, they are an important node between primary and secondary consumers within the trophic web (Thiele 1977, Boscaini et al. 2000, Kotze et al. 2011, Šerić Jelaska et al. 2013, Viterbi et al. 2013).

## General description

### Purpose

The main purpose of this dataset is to give open access to data as complete as possible on the distribution and abundance of carabid beetles in the Eastern Dolomites (Italian Alps).

### Additional information

**Figure 1.**
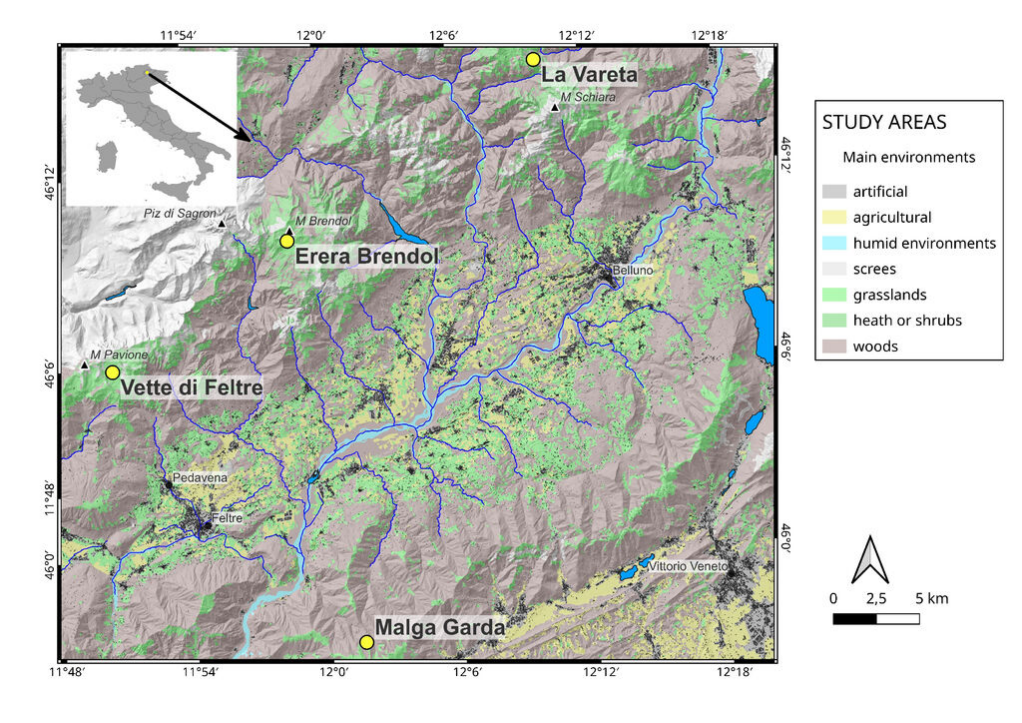
Location of the study areas (yellow dots).

This is the second available dataset, after Pizzolotto (2024) about the carabid fauna of the Eastern Dolomites. The dataset includes data collected from field research by Roberto Pizzolotto in the Vette di Feltre, La Vareta, Monte Garda and Erera Brendol mountains (see Fig. 1 for details). The data from Vette di Feltre 1983 and from La Vareta were published (in Italian) in Brandmayr and Pizzolotto (1989) and in Pizzolotto and Lasen (1997), respectively, while the rest of the data have never been published in the scientific literature.

## Sampling methods

### Sampling description

Carabid beetles were collected between 1983 and 1996, sampling 27 sample sites in different locations, in different years by pitfall traps. At the end of every sampling campaign, a “year’s sample” was obtained, which is the sum of the collections made during the research campaign. The pitfall traps were plastic vessels with an upper diameter of 9.2 cm, a depth of 11 cm and a small opening at 4 cm below the rim to avoid rainwater overflowing, filled with 200 ml of a preservative mixture made of wine vinegar and 5% formalin.

Given the unpredictable events that cause traps to break (e.g. cows, tourists, marmots), the number of individuals sampled in a year within each site (year sample) may be affected by an uneven sampling effort. For this reason, species abundance is evaluated as annual Activity Density (aAD), which is based on the total annual number of individuals caught and on the total annual sampling effort unit (EU), related to a period of ten days, as follows:

eu = (traps * days) / 10

eu is the effort unit for each single sampling period from which the total annual capture effort (EU) is given by the sum of:

EU = ∑eu

and, therefore,

aAD = total number of individuals / EU.

### Step description

Pitfall traps were randomly placed, distanced about 10 metres, in each of the sites included in the study (see Table 1), with a number ranging between four and six traps, which were emptied at an interval of about 25 days. Then, the samples were stored in alcohol and taken to the laboratory, where the carabid beetles were sorted, identified and counted. Finally, the data were described according to the Darwin Core standard and published through GBIF.

## Geographic coverage

### Description

The study area was in the South Eastern Dolomites, Italian Eastern Alps. The main peaks are (from west to east): Monte Pavione 2335 m, Piz di Sagron 2486 m, Monte Brendol 2160 m, Monte Talvena 2542 m and Monte Schiara 2565 m (see Fig. 1).

### Coordinates

45.95 and 46.26 Latitude; 11.83 and 12.171 Longitude.

## Taxonomic coverage

### Taxa included

**Table:** 

Rank	Scientific Name	Common Name
family	Carabidae	carabids

## Temporal coverage

**Data range:** 1983-6-07 – 1996-9-19.

## Usage licence

### Usage licence

Other

### IP rights notes

CC BY-NC 4.0

## Data resources

### Data package title

Carabid beetles in Eastern Dolomites from 1983 to 1996

### Resource link

https://doi.org/10.15468/tysq92

### Number of data sets

2

### Data set 1.

#### Data set name

Event

#### Data format

Darwin Core table (tab delimited)

#### Download URL

https://doi.org/10.15468/tysq92

#### Description

Table of the sampled sites. Twenty-seven sites (records) were sampled within 1300–2200 m altitude, including montane pastures to alpine scree-slopes. See text for details and map.

**Data set 1.** 

Column label	Column description
eventID	An identifier for the set of information associated with a dwc:Event (something that occurs at a place and time). Here an identifier specific to the dataset.
samplingProtocol	The names of, references to, or descriptions of the methods or protocols used during a dwc:Event.
sampleSizeValue	Number of active pitfall traps.
sampleSizeUnit	One pitfall trap.
samplingEffort	∑traps*(days/10).
eventDate	The date-time or interval during which a dwc:Event occurred.
startDayOfYear	The earliest integer day of the year on which the dwc:Event occurred (e.g. 33 is the 2nd of February).
endDayOfYear	The latest integer day of the year on which the dwc:Event occurred.
countryCode	The standard code for the country in which the dcterms:Location occurs.
maximumElevationInMetres	The upper limit of the range of elevation (altitude, usually above sea level), in metres.
habitat	A category or description of the habitat in which the dwc:Event occurred. Here the NAT2000 classification was used.
decimalLatitude	Latitude in decimal degrees.
decimalLongitude	Longitude in decimal degrees.
geodeticDatum	The ellipsoid, geodetic datum or spatial reference system (SRS), upon which the geographic coordinates given in dwc:decimalLatitude and dwc:decimalLongitude are based.
coordinateUncertaintyInMetres	The horizontal distance (in metres) from the given decimalLatitude and decimalLongitude describing the smallest circle containing the whole of the Location.
locality	Geographic information of the place where sample sites were located.

### Data set 2.

#### Data set name

Occurrence

#### Data format

Darwin Core table (tab delimited)

#### Download URL

https://doi.org/10.15468/tysq92

#### Description

Twenty-seven records (i.e. sample sites, see previous table) where the annual Activity Density of 66 species was given.

**Data set 2.** 

Column label	Column description
eventID	An identifier for the set of information associated with a dwc:Event (something that occurs at a place and time). Here an identifier specific to the dataset (the same as for the event table).
occurrenceID	An identifier for the Occurrence, possibly a persistent global unique identifier. Here, it is given by the eventID plus the four first letters of genus and species.
basisOfRecord	The specific nature of the data record. Here, all records come from human observation.
individualCount	The number of individuals present at the time of the dwc:Occurrence.
organismQuantity	A number or enumeration value for the quantity of organisms. Here, an index of activity density (see organismQuantityType column).
organismQuantityType	The type of quantification system used for the quantity of organisms. Here, the activity density = individualCount/sampling effort.
lifeStage	The age class or life stage of the dwc:Organism(s) at the time the dwc:Occurrence was recorded.
occurrenceStatus	A statement about the presence or absence of a dwc:Taxon at a Location.
scientificName	The full scientific name.
kingdom	The full scientific name of the kingdom in which the species is classified.
phylum	The full scientific name of the phylum in which the species is classified.
class	The full scientific name of the class in which the species is classified.
order	The full scientific name of the order in which the species is classified.
family	The full scientific name of the family in which the species is classified.
genus	The full scientific name of the genus in which the species is classified.
specificEpithet	The name of the species epithet of the dwc:scientificName.
taxonRank	The taxonomic rank of the most specific name in the dwc:scientificName.

## Additional information

A total of 56 species were collected in 27 sample sites (Table 2). Few of them (8) were present in more than a half of the sites, while the majority of them (37) were present in at least 10% of the sites. Six species were the most active (i.e. aAD > 10), while nearly half of them showed aAD less than 1.0. High frequency was not always linked to high aAD.

## Figures and Tables

**Table:** 

Nanopublication	Creator	Date
https://w3id.org/kpxl/pensoft/bdj/np/RAd4WSuE5TUHgAWd4HmqaS6VXUHlWAoGAg3eWDfrbqfqw	Roberto Pizzolotto	14-04-2025 11:20:25
https://w3id.org/kpxl/pensoft/bdj/np/RA6B7H9fktFKzdBYOtu5tXlO3GZJc7FVdRdoRSDw_R7og	Roberto Pizzolotto	14-04-2025 11:21:25
https://w3id.org/kpxl/pensoft/bdj/np/RAm2USHh5LYrg71rly9G3wvgH5pmtinesI_9_n6O4MfI4	Roberto Pizzolotto	14-04-2025 11:22:06
https://w3id.org/kpxl/pensoft/bdj/np/RA6wzz8dSN5Fx8tY03TZkbsSiZ-cLjGVBw_dpt6QCUKmA	Roberto Pizzolotto	14-04-2025 11:23:07

**Table 1.** Sample sites description. Data from sites with label ending with 1983 were published in Brandmayr and Pizzolotto (1989), while sites with label LaVar were published in Pizzolotto and Lasen (1997). The vegetation of the sites was determined on the basis of the NAT2000 habitats codes where possible (see European Commission (2013)). Aspect, Slope and Vegetation cover, were visually evaluated.

**Site**	**Altitude (a.s.l.)**	**Aspect**	**Slope**	**Veg. cover**	**Vegetation NAT2000**
VdFAo1_1983	1950 m	NE	45°	35%	8120 Calcareous and calcshist screes of the montane to alpine levels (*Thlaspietearotundifolii*)
VdFAo2_1983	2120 m	SSE	50°	40%	8120 Calcareous and calcshist screes of the montane to alpine levels (*Thlaspietearotundifolii*)
VdFAo3_1983	2080 m	WNW	45°	40%	8120 Calcareous and calcshist screes of the montane to alpine levels (*Thlaspietearotundifolii*)
VdFSC1_1983	2160 m	SSW	35°	95%	6170 Alpine and subalpine calcareous grasslands
VdFSC2_1983	2100 m	SW	45°	65%	6170 Alpine and subalpine calcareous grasslands
VdFSC3_1983	2080 m	NW	25°	50%	6170 Alpine and subalpine calcareous grasslands
VdFSC4_1983	2090 m	NE	40°	60%	6170 Alpine and subalpine calcareous grasslands
VdFSC5_1983	2090 m	W	20°	100%	6170 Alpine and subalpine calcareous grasslands
VdFAH_1983	1970 m	NNE	45°	60%	8120 Calcareous and calcshist screes of the montane to alpine levels (Thlaspietearotundifolii)
VdFAH2_1989	2000 m	NW	35°	60%	8120 Calcareous and calcshist screes of the montane to alpine levels (*Thlaspietearotundifolii*)
VdFRM_1989	1950 m	NW	40°	70%	4060 Alpine and Boreal heaths
VdFAd_1983	1980 m	E	35°	100%	8120 Calcareous and calcshist screes of the montane to alpine levels (*Thlaspietearotundifolii*)
VdFNa_1989	1880 m	NE	2°	100%	*Nardusstricta*-dominated grasslands
VdFRu_1989	1880 m	NNE	2°	80%	*Rumicetumalpini* on overgrazed pasture
VdFdia1_1983	2160 m	SSW	2°	50%	Rocky habitat
VdFdia2_1983	2050 m	SSE-NNW	2°	40%	Rocky habitat
ErBrEB1_1996	1950 m	SE	0°-2°	100%	Grassland in snow bed
ErBrEB2_1996	1960 m	SW	25°	100%	Grass mats on calcareous scree
ErBrEB3_1996	1970 m	NE	35°	1%	8120 Calcareous and calcshist screes of the montane to alpine levels (*Thlaspietearotundifolii*)
ErBrEB4_1996	1700 m	NW	25°	100%	Alpine pasture
LaVarLV1_1995	1700 m ca.	N	5°	100%	Mown grasslands and grazed pastures
LaVarLV2_1995	1700 m ca.	E	20°	100%	Mown grasslands and grazed pastures
LaVarLV3_1995	1700 m ca.	S	5°	100%	Mown grasslands and grazed pastures
LaVarLV4_1995	1700 m ca.	S	40°	90%	Mown grasslands and grazed pastures
LaVarLV5_1995	1700 m ca.	E	3°	100%	Mown grasslands and grazed pastures
MGarGa2_1990	1300 m	SE	20°	90%	Montane pastures
MGarGa1_1990	1300 m	NW	20°	100%	Montane pastures

**Table 2.** Species list. Frequence% gives the relative frequency, while Total aAD gives the sum of the aAD of each species in the 27 sample sites.

**Species**	**Frequence**%	**Total aAD**
*Pterostichusburmeisteri* Heer, 1837	100.0	81.288
*Pterostichusjosephi* Csiki, 1930	85.2	46.571
*Calathusmelanocephalus* (Linnaeus, 1758)	66.7	9.605
*Carabusgermarii* Sturm, 1815	66.7	7.269
*Carabuscreutzeri* Fabricius, 1801	63.0	17.499
*Trichotichnuslaevicollis* (Duftschmid, 1812)	63.0	10.500
*Carabusconvexus* Fabricius, 1775	55.6	4.124
*Amaraalpestris* A. & G.B.Villa, 1833	51.9	42.505
*Carabusbertolinii* (Kraatz, 1878)	48.1	6.996
*Notiophilusaquaticus* (Linnaeus, 1758)	44.4	1.117
*Abaxecchelii* Bertolini, 1887	37.0	5.997
*Duvaliusbreiti* (Ganglbauer, 1900)	33.3	1.026
*Nebriadiaphana* K. & J.Daniel, 1890	33.3	2.710
*Amaranitida* Sturm, 1825	29.6	1.587
*Cychrusangustatus* Hoppe & Hornschuch, 1825	29.6	0.520
*Leistusnitidus* (Duftschmid, 1812)	29.6	1.901
*Stomisrostratus* (Duftschmid, 1812)	29.6	0.319
*Abaxparallelepipedus* (Piller & Mitterpacher, 1783)	25.9	1.214
*Abaxpilleri* Csiki, 1916	25.9	8.997
*Calathusmicropterus* (Duftschmid, 1812)	25.9	1.540
*Tapinopterusplacidus* (Rosenhauer, 1847)	25.9	0.484
*Dyschiriusglobosus* (Herbst, 1784)	22.2	0.169
*Harpaluslatus* (Linnaeus, 1758)	22.2	0.834
*Harpalussolitaris* Dejean, 1829	22.2	0.654
*Molopspiceus* (Panzer, 1793)	22.2	0.864
*Trichotichnusknauthi* (Ganglbauer, 1901)	22.2	0.854
*Amaraerratica* (Duftschmid, 1812)	18.5	0.859
*Laemostenusjanthinus* (Duftschmid, 1812)	18.5	6.784
*Trechuspallidulus* Ganglbauer, 1891	18.5	2.508
*Cymindisvaporariorum* (Linnaeus, 1758)	14.8	0.439
*Pterostichusschaschli* (Marseul, 1880)	14.8	1.900
*Amaramontivaga* Sturm, 1825	11.1	0.170
Bradycellus caucasicus (Chaudoir, 1846)	11.1	0.044
*Harpalusrubripes* (Duftschmid, 1812)	11.1	0.101
*Lebiachlorocephala* (J.J.Hoffmann, 1803)	11.1	0.074
*Notiophilusbiguttatus* (Fabricius, 1779)	11.1	0.432
*Poecilusversicolor* (Sturm, 1824)	11.1	7.293
*Amaracommunis* (Panzer, 1796)	7.4	0.123
*Amaralunicollis* Schiödte, 1837	7.4	2.976
*Bembidionglaciale* Heer, 1837	7.4	0.524
*Bembidionlampros* (Herbst, 1784)	7.4	11.243
*Cychrusattenuatus* (Fabricius, 1792)	7.4	1.585
*Laemostenusschreibersii* (Küster, 1846)	7.4	0.041
*Amarabrunnea* (Gyllenhal, 1810)	3.7	3.500
*Badisterbullatus* (Schrank, 1798)	3.7	0.013
*Bembidiondeletum* Audinet-Serville, 1821	3.7	0.010
*Carabuscoriaceus* Linnaeus, 1758	3.7	0.118
*Carabusproblematicus* Herbst, 1786	3.7	0.013
*Harpalushonestus* (Duftschmid, 1812)	3.7	0.027
*Harpaluslaevipes* Zetterstedt, 1828	3.7	0.042
*Harpalusmarginellus* Gyllenhal, 1827	3.7	0.056
*Nebriajockischii* Sturm, 1815	3.7	0.020
*Pterostichusrhaeticus* Heer, 1837	3.7	0.020
*Pterostichusstrenuus* (Panzer, 1796)	3.7	0.048
*Pterostichusvernalis* (Panzer, 1796)	3.7	0.056
*Synuchusvivalis* (Illiger, 1798)	3.7	0.143
